# Immuno-PET Imaging of HER3 in a Model in which HER3 Signaling Plays a Critical Role

**DOI:** 10.1371/journal.pone.0143076

**Published:** 2015-11-16

**Authors:** Qinghua Yuan, Takako Furukawa, Takahiro Tashiro, Kouki Okita, Zhao-Hui Jin, Winn Aung, Aya Sugyo, Kotaro Nagatsu, Hiroko Endo, Atsushi B. Tsuji, Ming-Rong Zhang, Takashi Masuko, Masahiro Inoue, Yasuhisa Fujibayashi, Tsuneo Saga

**Affiliations:** 1 Molecular Imaging Center, National Institute of Radiological Sciences, Chiba, Japan; 2 Department of Biochemistry, Osaka Medical Center for Cancer and Cardiovascular Diseases, Osaka, Japan; 3 Cell Biology Laboratory, Department of Pharmaceutical Sciences, School of Pharmacy, Kinki University, Higashi Osaka, Japan; 4 Carna Biosciences Inc., Kobe, Japan; Alexion Pharmaceuticals, UNITED STATES

## Abstract

HER3 is overexpressed in various carcinomas including colorectal cancer (CRC), which is associated with poor prognosis, and is involved in the development of therapy resistance. Thus, an *in vivo* imaging technique is needed to evaluate the expression of HER3, an important therapeutic and diagnostic target. Here, we report successful HER3 PET imaging using a newly generated anti-human HER3 monoclonal antibody, Mab#58, and a mouse model of a HER3-overexpressing xenograft tumor. Furthermore, we assessed the role of HER3 signaling in CRC cancer tissue-originated spheroid (CTOS) and applied HER3 imaging to detect endogenous HER3 in CTOS-derived xenografts. Cell binding assays of ^89^Zr-labeled Mab#58 using the HER3-overexpressing cell line HER3/RH7777 demonstrated that [^89^Zr]Mab#58 specifically bound to HER3/RH7777 cells (K_*d*_ = 2.7 nM). *In vivo* biodistribution study in mice bearing HER3/RH7777 and its parent cell xenografts showed that tumor accumulation of [^89^Zr]Mab#58 in HER3/RH7777 xenografts was significantly higher than that in the control from day 1 to day 4, tending to increase from day 1 to day 4 and reaching 12.2 ± 4.5%ID/g. Radioactivity in other tissues, including the control xenograft, decreased or remained unchanged from day 1 to day 6. Positron emission tomography (PET) in the same model enabled clear visualization of HER3/RH7777 xenografts but not of RH7777 xenografts. CTOS growth assay and signaling assay revealed that CRC CTOS were dependent on HER3 signaling for their growth. In PET studies of mice bearing a CRC CTOS xenograft, the tumor was clearly visualized with [^89^Zr]Mab#58 but not with the ^89^Zr-labeled control antibody. Thus, tumor expression of HER3 was successfully visualized by PET with ^89^Zr-labeled anti-HER3 antibody in CTOS xenograft-bearing mice, a model that retains the properties of the patient tumor. Non-invasive targeting of HER3 by antibodies is feasible, and it is expected to be useful for cancer diagnosis and treatment.

## Introduction

HER3 is a member of the epidermal growth factor receptor (EGFR) family. Each of the four family members, EGFR (erbB1 or HER1), HER2 (erbB2), HER3 (erbB3), and HER4 (erbB4) contains a large extracellular (ligand-binding) domain, a single membrane-spanning region, and a cytoplasmic (protein TK) domain [1]. This family is involved in epithelial cell differentiation, growth, division and motility, and alteration or disruption of their function plays important roles in the development and progression of malignancy [2,3]. HER3 is unique among the family members because it contains a truncated intracytoplasmic domain that is deficient in TK activity [4–6] and depends on heterodimer formation, usually with HER2, to mediate its signaling activity [7,8]. HER3 is overexpressed in many carcinomas, including colorectal cancer (CRC), which is associated with poor prognosis [9,10], making it a target of cancer therapy and diagnosis.

The importance of HER3 as a therapeutic target has garnered considerable attention because it was revealed that resistance to HER-family tyrosine kinase inhibitor therapy depends on HER3 signaling pathways [11,12], and that HER3 is involved in the development of resistance against chemotherapy [13]. Anti-HER3 antibodies have been developed for therapeutic use [14,15], with some anti-HER3 antibodies being reported to abrogate resistance against agents targeting the EGFR family in CRC and breast cancer cells [16,17]. Some studies also report the in vivo imaging of HER3 [18–21].

Cancer Tissue-Originated Spheroid (CTOS) is a recently developed tissue culture method, in which the properties of original tumors are preserved by maintaining cell-cell contact [22]. CTOSs can be prepared from various types of cancers including colorectal, urothelial, and lung cancers and are expected to provide a unique and valuable model for cancer research [22–24]. The structure of the spheroid, unlike that of a monolayer culture, provides an opportunity to explore the factors critical for malignant progression of cancer, possibly relating to invasion or metastasis, in the context of a three-dimensional (3D) structure, which partly mimics the in vivo tumor conditions and shares a strong similarity with patient tumors. Moreover, CTOS-derived xenograft tumors resemble original patient tumors in terms of 3D structure as well as gene expression [22]. Therefore, CTOS-derived xenografts provide a better platform for the preclinical evaluation of imaging probes.

Here, we report the feasibility of HER3 PET imaging in vivo by using a newly generated anti-human HER3 monoclonal antibody with mouse tumor models of a HER3-overexpressing cell line. Furthermore, as our previous works have revealed that HER3 signaling plays an important role in the growth of lung and urothelial cancer CTOSs [23,24], we assessed the role of HER3 signaling in CRC CTOS, and applied the HER3 imaging technique to detect endogenous HER3 in CTOS-derived xenografts.

## Materials and Methods

### Ethics Statement

The protocol for CTOS experiments was approved by the ethics committees of Osaka Medical Center for Cancer and Cardiovascular Diseases and the National Institute of Radiological Sciences. The animal experimental protocol was approved by the Animal Care and Use Committee of the National Institute of Radiological Sciences (permit number: 07-1064-19), and all animal experiments were conducted in accordance with the institutional guidelines regarding animal care and handling. All efforts were made to minimize suffering of the animals in all the experiments.

### Cells and CTOSs

HER3/RH7777, a cell line stably overexpressing human HER3 linked to green fluorescent protein established from the rat hepatoma cell line RH7777, and the parent cell line were kindly provided by Dr. Chiba (Mitsubishi Tanabe Pharma, Osaka, Japan). The cells were maintained in Dulbecco’s modified Eagle’s medium (DMEM, 11995–065; Invitrogen, Thermo Fisher Scientific, Waltham, MA) supplemented with 10% fetal bovine serum, 50 U/mL penicillin, and 50 μg/mL streptomycin (Gibco, Carlsbad, CA) in a humidified incubator maintained at 37°C with 5% CO_2_. For HER3/RH7777 culture media, G418 (400 ng/mL, Gibco) was also added.

C45, a CTOS derived from a patient diagnosed with a moderately differentiated colon adenocarcinoma, was prepared and maintained as described previously [22,23]. The CTOSs were cultured in StemPro hESC medium (Invitrogen) supplemented with 8 ng/mL bFGF (Invitrogen), 0.1 mM β-mercaptoethanol (Wako, Osaka, Japan), and antibiotics in a non-treated dish (IWAKI, Tokyo, Japan) in a humidified atmosphere at 37°C with 5% CO_2_.

### CTOS growth assay

CTOSs were embedded in Matrigel Matrix Growth Factor Reduced (GFR) (BD Biosciences, Bedford, MA) and cultured in 100 μL of StemPro hESC or basal medium containing one of the following growth factors: 10 ng/mL of NRG 1 (PeproTech, Rocky Hill, NJ), 200 ng/mL of Long-IGF1 (GroPep, Adelaide, Australia), 8 ng/mL of bFGF, or 10 ng/mL of Activin A (R&D Systems, Minneapolis, MN). Basal medium consisted of DMEM/F12, 1x GlutaMAX, 0.1 mM 2-mercaptoethanol, 2% BSA, 50 U/mL penicillin, and 50 μg/mL streptomycin. CTOS growth was evaluated by the CTOS area at the indicated time point, corrected for the CTOS area at day 0. CTOS area was measured using an image analysis software (Image J, NIH).

### Signal assay in CTOS

C45 CTOS were prepared from mouse xenografts as previously described [22]. After culture for 1 day, the medium was changed from StemPro hESC to starvation medium, the basal medium mentioned above. After an overnight culture in the starvation medium, CTOS were stimulated by 10 ng/mL of NRG 1 or 1/50 volume of StemPro supplement. After 15 min, the cells were subjected to western blotting, as previously described [23].

### Subcutaneous tumor models

BALB/c-nu/nu female mice (aged 5–6 weeks; CLEA Japan, Tokyo, Japan) were maintained under pathogen-free conditions. For the cell line tumor model, mice were inoculated subcutaneously with HER3/RH7777 (1 × 10^6^) and RH7777 (1 × 10^6^) cells in the left and right thighs, respectively, under isoflurane anesthesia. The mice were used for biodistribution and PET study at about 2 weeks after inoculation, when the tumors were 5–10 mm in diameter. For CTOS tumor model, approximately 1000 CTOS C45s of diameter 40–100 μm were suspended in 50 μL of Matrigel Matrix GFR (BD Biosciences) and transplanted subcutaneously into the flanks of NOD/SCID mice (NOD.CB17-Prkdc SCID /J, Charles River Japan, Yokohama, Japan). The CTOS tumor-bearing mice were used for further experiments once the tumor diameter reached 10–15 mm, which occurred 3–6 weeks after transplantation. General conditions of the mice were monitored at least twice a week.

### HER3 protein expression in xenografts

Xenograft tumors of HER3/RH7777, RH7777, and CTOS C45 were excised and fixed in neutralized 10% formalin, embedded in paraffin, and cut into 4-μm-thick sections. The sections were incubated with diluted mouse anti-HER3 monoclonal antibody (2.5 μg/mL; erbB3/Her3, nanoTools, Teningen, Germany) for 1 h at room temperature, washed, and then incubated with peroxidase-conjugated secondary antibodies (EnVision+ System-HRP Labeled Polymer Anti-mouse; DAKO, Glostrup, Denmark). The binding of the secondary antibodies was visualized with diaminobenzidine staining (K3465; DAKO) and counterstaining was carried out with hematoxylin.

### Preparation of rat monoclonal antibodies recognizing HER3

Female F344 rats were administered subcutaneously and intraperitoneally with HER3/RH7777 cells in the first immunization, followed by four booster intraperitoneal and intravenous injections of HER3/RH7777 cells at a 10-day interval. Three days after the final immunization, spleen cells of immunized rats were isolated and fused with P3X63Ag8.653 mouse myeloma cells by using 50% polyethylene glycol 1540 (Roche Diagnostics, Tokyo, Japan). After the cell fusion, hybridoma cells were selected in 7% FBS-containing RPMI1640 medium (Sigma-Aldrich, Tokyo, Japan) supplemented with hypoxanthine, aminopterin, and thymidine (HAT supplement; Life Technologies, Tokyo, Japan). Antibodies secreted from hybridoma clones were selected for their reactivity against HER3/RH7777 cells in a GFP expression-dependent manner [25, 26]. Anti-HER3 mAb (Mab#58, IgG2a) was also confirmed to show no reactivity with RH7777 or HEK293 cells expressing HER1, HER2, or HER4 proteins. Anti-mouse CD44v rat mAb [27, 28] was used as control IgG for Mab#58 in this study.

### Radiolabeling of antibodies

Mab#58, and the control antibody were conjugated with *p*-isothiocyanatobenzyl-desferrioxamine B (DF; Macrocyclics, Dallas, TX, USA) at DF at an antibody molar ratio of 3:1, as previously described [29]. The conjugation ratio of DF to antibody was estimated to be 2.3:1 to 2.5:1, as determined by size exclusion chromatography using a PD10 column (GE Healthcare, Buckinghamshire, UK) before purification. Non-conjugated chelate was removed using a Sephadex G-50 (GE Healthcare) spin column. ^89^Zr was produced using the NIRS AVF-930 cyclotron, and ^89^Zr-oxalate (3.7–5.6 GBq/mL in 1 M oxalate, pH 7–8) was prepared as described previously [29]. The DF-conjugated antibodies (5 mg/mL in PBS) was incubated with 1/5 volume of ^89^Zr-oxalate solution for 1 h at room temperature, and radiolabeled antibodies were purified on a Sephadex G-50 spin column. The radiochemical purity was over 90%, and the specific radioactivity was 40–110 kBq/μg, as determined by thin-layer chromatography, using 50 mM diethylenetriaminepentaacetic acid, pH 7, as the mobile phase.

### *In vitro* assay of the labeled antibodies

Cell binding, competitive inhibition, and internalization assays were conducted as previously described [29]. In the cell binding assay, HER3/RH7777 and RH7777 cells in Dulbecco’s phosphate-buffered saline (D-PBS(-); WaKo) with 1% BSA (Bovine Serum Albumin, Gibco) were incubated with the ^89^Zr labeled Mab#58 ([^89^Zr]Mab#58) on ice for 60 min. After washing with cold D-PBS(-) twice, the radioactivity of [^89^Zr]Mab#58 bound to the cells was measured using a gamma counter. From the cell binding assay, immunoreactive fraction was calculated by the method of Lindmo et al [30]. In the competitive inhibition assay, [^89^Zr]Mab#58 was incubated with HER3/RH7777 cells in the presence of varying concentrations of the unlabeled Mab#58, unconjugated or DF-conjugated, for 1 h on ice. After washing, the radioactivity bound to the cells was measured. The equilibrium dissociation constant was estimated using GraphPad Prism software (GraphPad Software, La Jolla, CA, USA), and the maximum binding capacity was calculated through Scatchard plot analysis. In the internalization assay, HER3/RH7777 cells were preincubated in culture medium with [^89^Zr]Mab#58 on ice for 60 min. After washing with cold D-PBS(-) twice, the cells were collected and further incubated at 37°C or on ice in fresh medium for various periods, and the supernatant and the cells were separated by centrifugation. The supernatant was incubated with trichloroacetic acid for 15 min on ice and then centrifuged to separate the non-protein-bound (supernatant) and protein-bound (pellet) fractions. The cells were washed with acidic buffer (0.05M glycine-HCl buffer, pH 2.8, containing 0.1M NaCl) and then separated by centrifugation to determine both the membrane-bound (supernatant) and internalized (pellet) fractions. The in vitro assays were conducted in duplicate.

### Biodistribution studies

Mice bearing both HER3/RH7777 and RH7777 tumors were injected with 148 kBq [^89^Zr]Mab#58 (approximately 2.5 μg protein) via the tail vein. At 1, 2, 4, and 6 days post-injection, five mice at each time point were euthanized by isoflurane inhalation, and their blood was obtained from the heart. Tumors and major organs were dissected and weighed, and radioactivity was measured using a gamma counter with decay correction. Radioactivity concentration was expressed as a percentage of the injected dose per g of tissue (%ID/g) normalized to a body weight of 20 g. Data are expressed as mean ± SD.

### PET imaging

Three mice bearing both HER3/RH7777 and RH7777 tumors were injected with approximately 3.7 MBq of [^89^Zr]Mab#58 (approximately 60 μg protein) via the tail vein. At 1, 2, 4, and 6 days post-injection, PET data acquisition was conducted for 10–20 min by using a small-animal PET system (Inveon, Siemens Medical Solutions, Malvern, PA) under isoflurane anesthesia during the entire scanning period. Images were reconstructed using a 3D maximum *a posteriori* (MAP) method (18 iterations with 16 subsets, *β* = 0.2) without attenuation correction. Image analysis was performed using the ASIPro VM Micro PET Analysis software (Siemens, Knoxville, TN). A lamp and heating pad were used during the PET scan to maintain the body temperature of the mice. Tracer uptake was expressed as % ID/g. The region of interest was manually drawn over tumors and tracer uptake was quantified using ASI Pro software (Siemens Medical Solutions). No correction for partial volume effects was performed.

Three mice bearing the CTOS C45 xenograft were injected with 3.7 MBq of [^89^Zr]Mab#58 or ^89^Zr labeled control antibody ([^89^Zr]IgG), and PET data were acquired as mentioned above.

### Statistical analysis

Statistical analysis was performed using one-way ANOVA, followed by Bonferroni test and *t*-text, using SPSS 12.0 (SPSS Inc., Chicago, IL).

## Results

### Feasibility of Immuno-PET imaging of HER3 by [^89^Zr]Mab#58

#### HER3 protein expression in the xenograft of a HER3 overexpressing cell line

**Fig 1:**
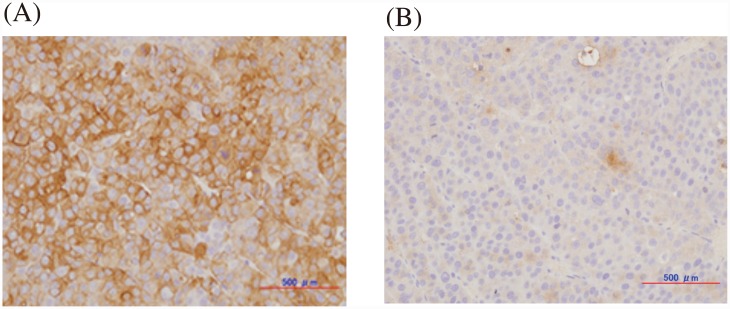
Representative immunohistochemical staining of HER3/RH7777 (A) and RH7777 (B) xenografts for HER3 expression.

The representative immunostaining images of HER3 overexpressing HER3/RH7777 and the parent RH7777 xenograft tumors are shown in Fig 1A and 1B, respectively. Almost all cells in the HER3/RH7777 tumor were positively stained for HER3, while few cells were stained in the RH7777 tumor, confirming the overexpression of HER3 in vivo and the applicability of the xenograft model for further studies.

#### *In vitro* assay of labeled antibody

**Fig 2:**
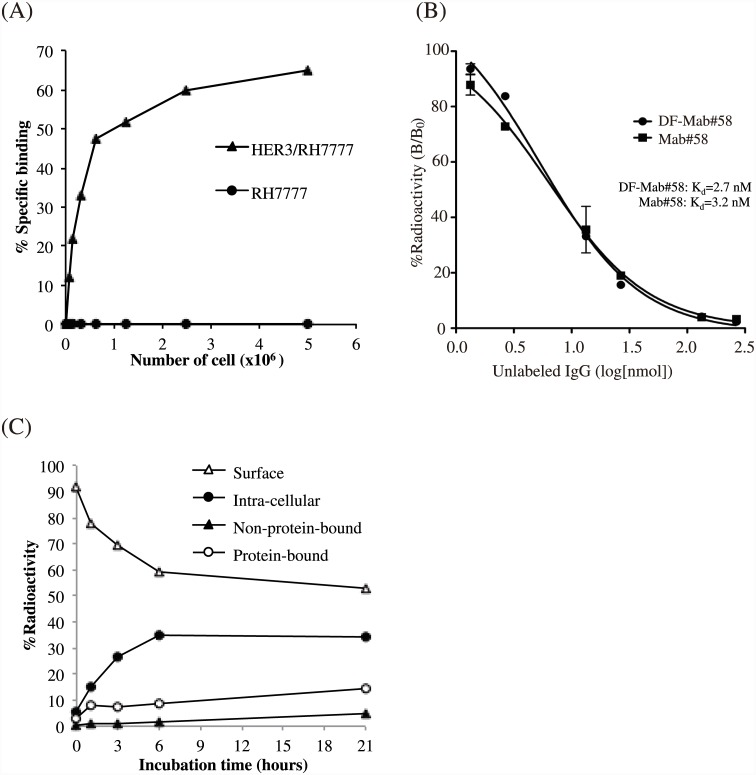
*In vitro* assays of radiolabeled anti-HER3 antibody Mab#58. (A) Cell binding assay for [^89^Zr]Mab#58 comparing HER3 overexpressing HER3/RH7777 cells (closed triangles) and parent RH7777 cells (closed circles). (B) Competitive inhibition assay for DF-conjugated (closed circles) and unconjugated Mab#58 (closed squares). (C) Internalization assay for [^89^Zr]Mab#58 at 37°C. Changes in the percentage of total radioactivity for each fraction are plotted against incubation time at 37°C (closed circles, internalized fraction; open triangles, membrane-bound fraction; open circles, protein-bound fraction in the culture medium; closed triangles, non-protein-bound fraction in the culture medium). These assays were conducted in duplicate. Data represent the mean.

As shown in Fig 2A, binding of [^89^Zr]Mab#58 to HER3/RH7777 cells increased with cell number, reaching 65% at a cell density of 5 × 10^6^ cells; however, binding to RH7777 cells was negligible, indicating HER3-specific binding of the labeled antibody. The immunoreactive fraction of [^89^Zr]Mab#58 was 73.1%. The dissociation constants (K_*d*_) of DF-conjugated and unconjugated Mab#58 calculated from the competitive binding assay were 2.67 and 3.16 nM, respectively (Fig 2B), and the maximum binding capacity of HER3/RH7777 for DF-conjugated and unconjugated Mab#58 was 3.1 x 10^5^ binding site/cell and 3.4 x 10^5^ binding site/cell, respectively. Approximately 34% of the [89Zr]Mab#58 that initially bound to the cell surface was internalized during the 21-h incubation at 37°C, and most of the internalized radioactivity was retained within the cells during the examination period. The radioactivity released (protein bound and unbound) was approximately 20% of the total radioactivity that initially bound to the cells, and 53% of the total radioactivity was bounded to the cell surface even after a 21-h incubation at 37°C (Fig 2C). When the cells were incubated on ice, the membrane bound fraction did not internalized or released for up to a minimum of 3 h (data not shown).

#### Biodistribution of [^89^Zr]Mab#58 in mice bearing HER3 overexpressing tumor

**Fig 3:**
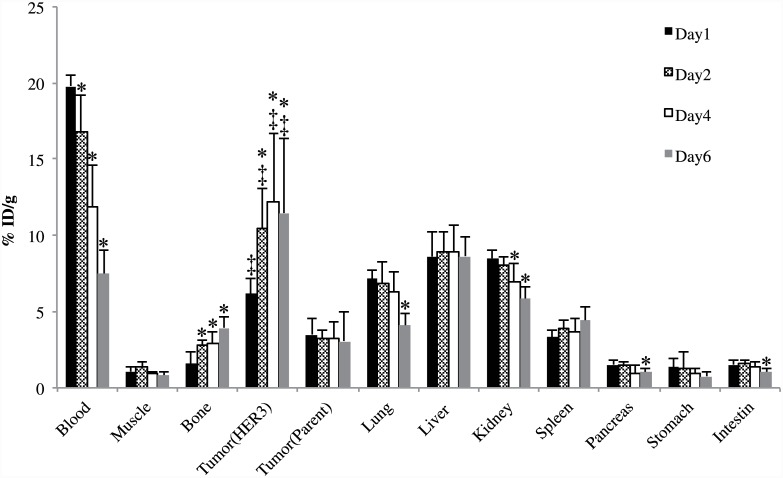
*In vivo* biodistribution study of [^89^Zr]Mab#58 in nude mice bearing both HER3 overexpressing HER3/RH7777 and parent RH7777 xenografts. Samples were collected and weighted, and the radioactivity was measured at day 1 (black bars), 2 (patterned bars), 4 (white bars) and 6 (gray bars) after intravenous injection of 37 kBq each of [^89^Zr]Mab#58. Data are expressed as mean ± SD (n = 5). *: significantly different from day 1 time points (p<0.05). ‡: significantly higher than parent RH7777 tumor.

Biodistribution of [^89^Zr]Mab#58 in nude mice bearing both HER3/RH7777 and RH7777 xenograft tumors is shown in Fig 3. The radioactivity (%ID/g) in the HER3/RH7777 xenograft 2–6 days after the injection of the labeled antibody was higher than that on day 1; it tended to increase from day 1 to day 4, reaching 12.2±4.5%ID/g at day 4. In contrast, radioactivity in most tissues examined, including the RH7777 xenograft, decreased or remained unchanged from day 1 to day 6 (Fig 3). The radioactivity in the bone slightly increased with time. The radioactivity (%ID/g) in HER3/RH7777 xenograft was significantly higher than that in RH7777 xenograft through day 1 to day 6, indicating specific accumulation of the labeled antibody.

#### PET imaging of mice bearing HER3 overexpressing tumor

**Fig 4:**
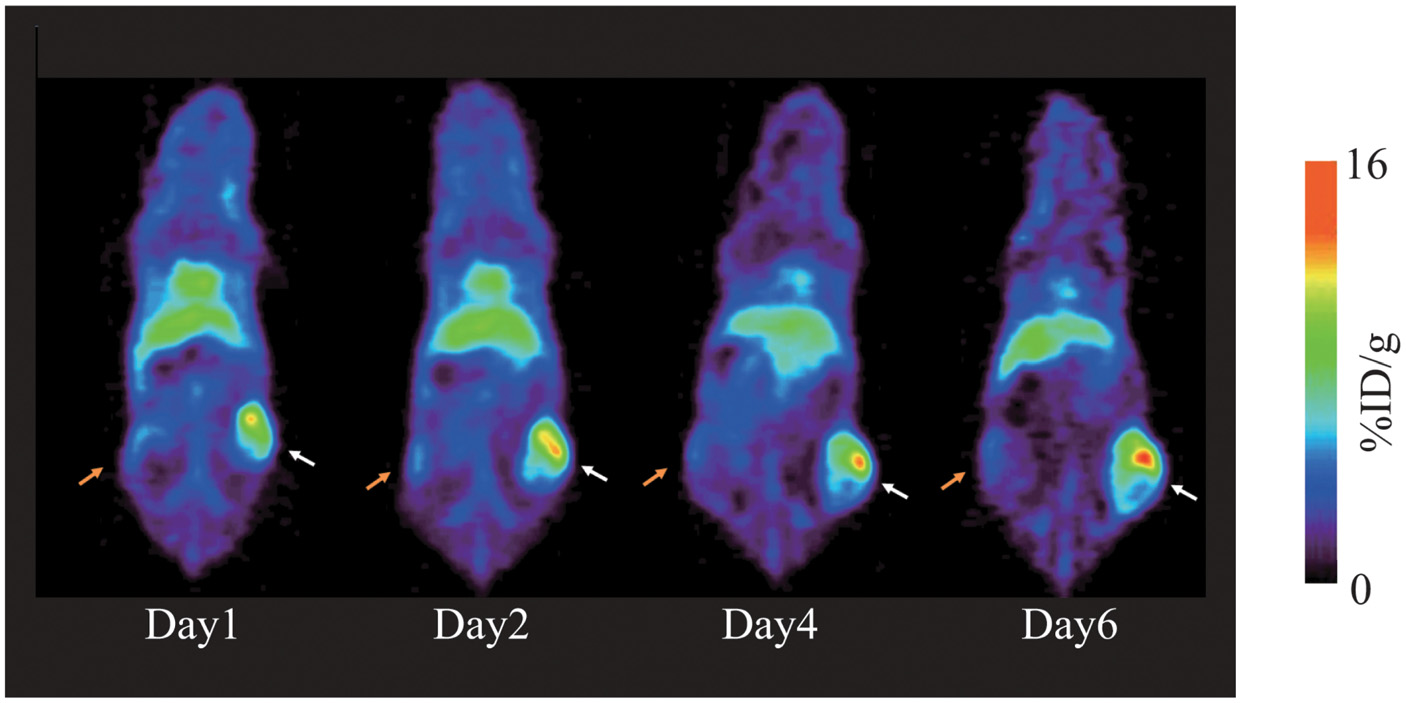
Representative PET images of mice bearing both HER3 overexpressing HER3/RH7777 and parent RH7777 xenografts. Serial PET images (maximum-intensity-projection (MIP)) of a mouse bearing HER3/RH7777 (right thigh, white arrow) and RH7777 (left thigh, orange arrow) tumors at days 1, 2, 4, and 6 after an intravenous injection of 3.7 MBq [^89^Zr]Mab#58.

Representative small animal PET images of a mouse bearing HER3 overexpressing HER3/RH7777 (right thigh) and parent RH7777 (left thigh) tumors at 1, 2, 4, and 6 days after [^89^Zr]Mab#58 administration are shown in Fig 4. The HER3/RH7777 xenograft was clearly visualized, with the accumulation apparently higher than that in the RH7777 xenograft, from day1 (13.6% ID/g) to day 6 (15.5% ID/g), without correction for partial volume effects (tumor volume: 130–310 mm^3^). The radioactivity in RH7777 tumor and the most of other tissues decreased from day 1 to day 6, confirming the results of the biodistribution study.

### HER3 in a CRC CTOS and the PET imaging

#### Effect of HER3 signaling on the growth of CRC CTOSs

**Fig 5:**
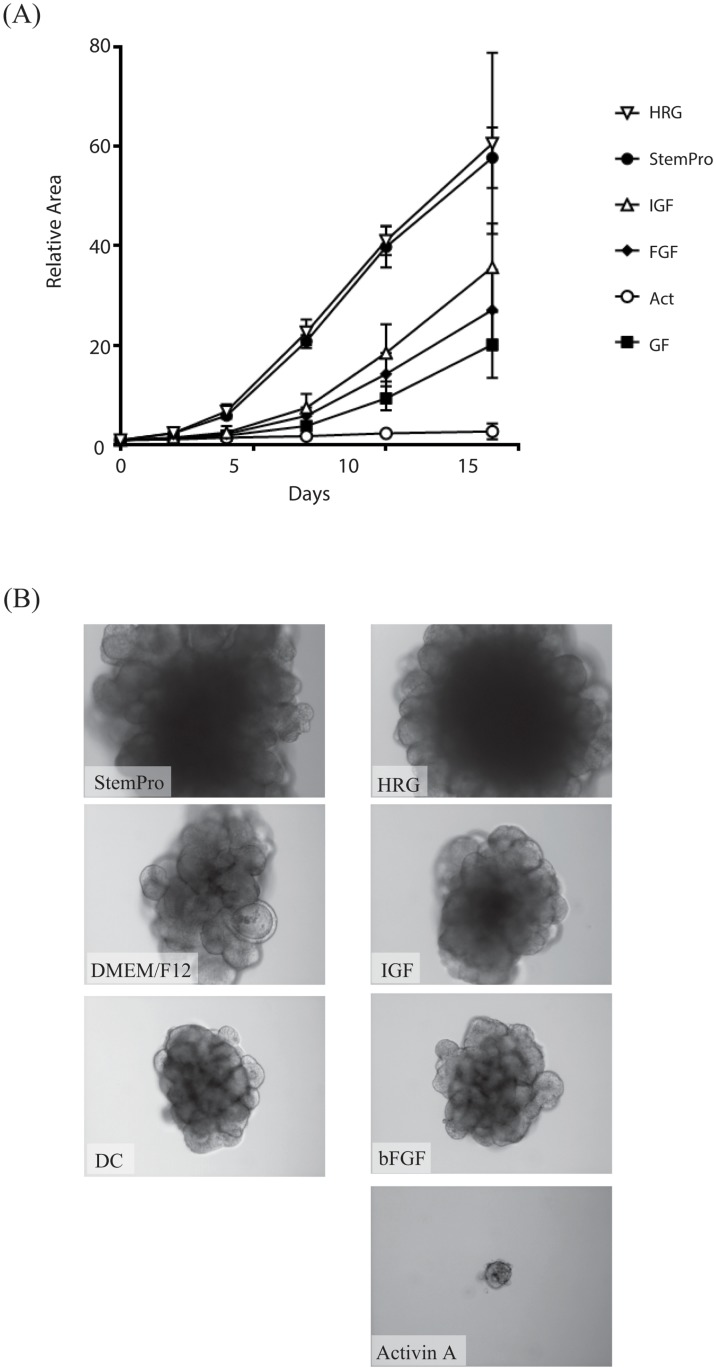
CTOS growth in various culture conditions. (A) Growth curve of CTOS cultured in StemPro hESC (Invitrogen) or basal medium containing one of the growth factors as indicated. (B) Representative images of CTOS at day 14 in (A).

Based on our previous observation that a culture medium for human ES cells is the most suitable for culturing CRC CTOSs [22], we investigated the growth factors contributing to the growth of C45 CRC CTOSs. Among the growth factors in the medium, heregulin (HRG) alone was enough to substitute the full medium, while other growth factors had a much lesser effect on the growth of C45 CTOSs (Fig 5A and 5B).

#### Activation of HER3 signaling by its ligand in CTOS

**Fig 6:**
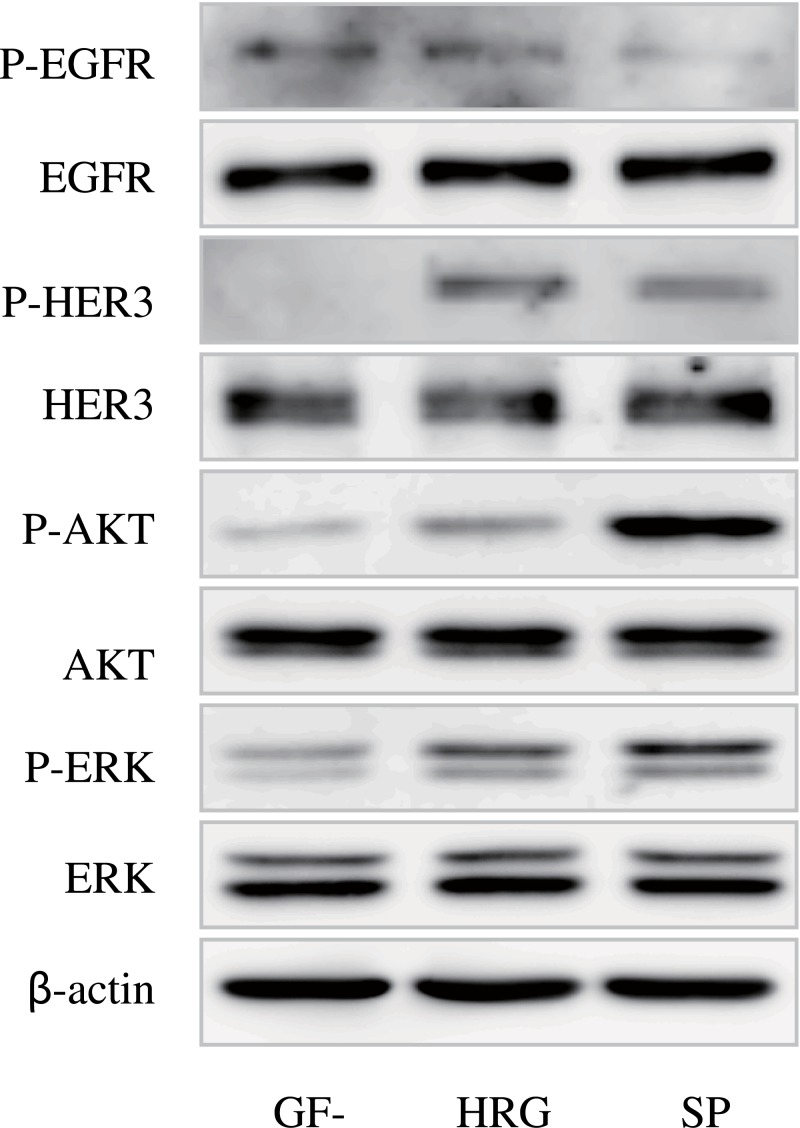
Western blotting of the signaling molecules in C45 in growth factor free conditions (GF-) or after stimulation with HRG or StemPro hESC medium (SP). Antibodies used are indicated. P-: antibodies against each phosphorylated molecule.

In parallel with growth stimulation, HER3 signaling was stimulated by a growth factor cocktail of human ES cell medium as well as HRG alone (Fig 6). While HER3 and downstream molecules including AKT and ERK were remarkably phosphorylated by both media, EGFR was not phosphorylated. Thus, C45 CTOS was found to be dependent on HER3 signaling for its growth.

#### HER3 protein expression in CTOS xenograft

**Fig 7:**
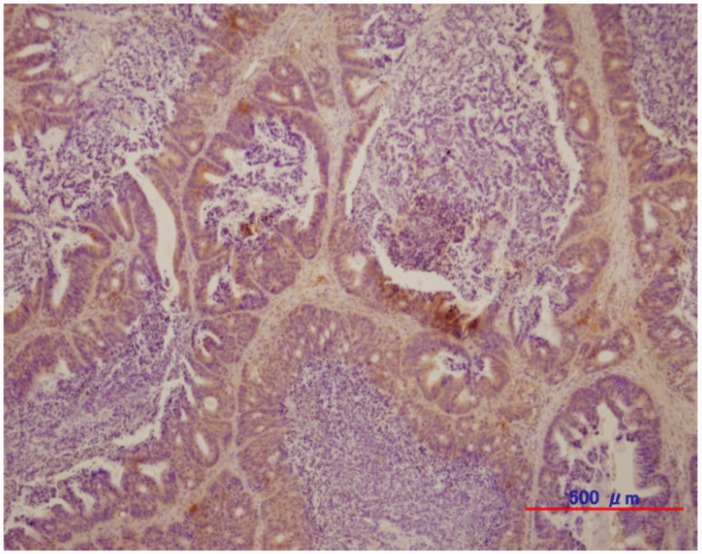
Representative immunohistochemical staining of CTOS C45 xenograft for HER3 expression.

Fig 7 shows the HER3 immunostaining of the CTOS C45 xenograft. Most cancer cells forming glandular structures in CTOS C45 tumor were positive for HER3, indicating that the endogenous levels of HER3 were high in the tumor.

#### PET imaging of CTOS derived tumor-bearing mice

**Fig 8:**
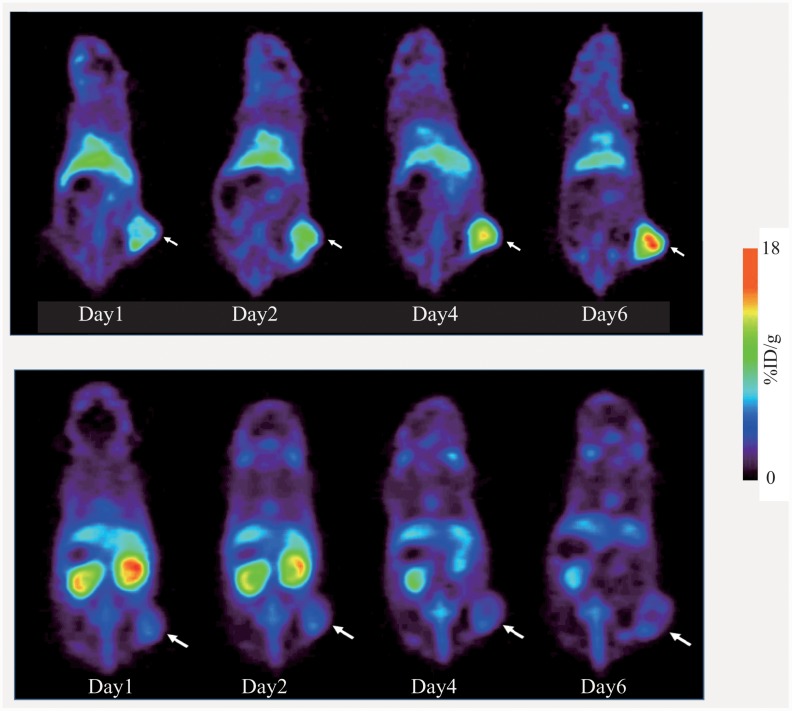
Representative PET images of mice bearing CTOS C45 xenografts. Serial PET images of mice bearing C45 xenograft (white arrow) injected with [^89^Zr]Mab#58 (upper panels) or similarly ^89^Zr-labeled control IgG.

Fig 8 shows the representative PET images of CTOS C45 xenograft-bearing mice. In the mouse administered [^89^Zr]Mab#58 (upper panels), the tumor was clearly visualized, with radioactivity in the tumor increasing from day1 (11.1% ID/g) to day6 (17.8% ID/g), without correction for partial volume effects (tumor volume: 170–350 mm^3^), while the accumulation of radioactivity in the tumor was low in the mouse administered [^89^Zr]IgG (lower panels), with radioactivity in the tumor decreasing from day1 (6.12% ID/g) to day 6 (5.5% ID/g). In the mice administered with [^89^Zr]IgG, high accumulation of radioactivity was observed in the kidney, which decreased with time (lower panels).

## Discussion

Here, we labeled an anti-HER3 monoclonal antibody Mab#58 with ^89^Zr (T_1/2_ = 78.4h) and evaluated its in vitro and in vivo properties using the HER3 overexpressing cell line HER3/RH7777, its parent cell line RH7777, and the mice bearing their xenografts. Human HER3 was expressed at a high level in the HER3/RH7777 xenograft, while the RH7777 xenograft was barely stained with anti-human HER3 antibody, making the mouse bearing the two xenografts a good model with clear contrast between the two grafts.

In vitro characterization showed that the binding of [^89^Zr]Mab#58 to HER3-expressing cells was specific, because its binding to the parent RH7777 cells not expressing human HER3 was found to be negligible, and the affinity of [^89^Zr]Mab#58 was high (K_*d*_ = 3.2 nM). The conjugation of DF had little effect on the affinity, despite of the conjugation level (DF/IgG) being higher than those reported for the same reaction condition earlier [29,31], and the immunoreactive fraction was fairly preserved. This confirmed that the conjugated antibody was suitable for further studies. The majority of [^89^Zr] radioactivity was retained with the cells after the binding of [^89^Zr]Mab#58 to the cells in vitro, with only 5% of the cell-bound radioactivity released as protein-free fraction and 10% as a protein-bound fraction in 21 h of incubation at 37°C. Accordingly, less than 20% of total radioactivity initially bound to the cells was released. The stable binding of [^89^Zr]Mab#58 to cells provides an advantage in high tumor accumulation in vivo.

The higher accumulation of [^89^Zr]Mab#58 in the HER3/RH7777 xenograft than in RH7777 xenograft throughout the time of examination indicated the HER3-specific accumulation. The PET imaging of the mice with HER3/RH7777 and RH7777 xenografts confirmed the result of our biodistribution analysis with a clear contrast between the two xenografts. The results, in vitro and in vivo, demonstrated the feasibility of in vivo imaging specific to HER3 expression with [^89^Zr]Mab#58.

Elevated HER3 expression levels in the patient’s pathological samples were suggested to be a prognostic marker as it is associated with poor prognosis, such as decreased time to disease progression [14,15]. Furthermore, HER3 gene expression is implicated to be a predictive biomarker for indicating the lack of benefit from cetuximab, an anti-EGFR antibody [32]. Heregulin, a ligand of HER3, also mediates cetuximab resistance [16]. Phase I clinical trials for solid tumors including CRC were accomplished with a monoclonal anti-HER3 antibodies, in which some of the patients showed stable disease progression [33,34]. Thus imaging of HER3 would be useful for developing a treatment strategy in a CRC patient.

Importance of HER3 in CRC progression was revealed by a preclinical study that used conventional CRC cell lines [14]. Here, we revealed that a CRC CTOS C45, which is directly derived from the patient and cultured in 3D conditions, was dependent on HER3 signaling for growth.

Most cancer cells in the C45 CTOS xenograft were positive for HER3 expression, suggesting an active role of HER3 in an in vivo setting. In the PET study with C45 xenografts, HER3 specificity was demonstrated by the differential accumulation pattern of [^89^Zr]Mab#58 and the control [^89^Zr]IgG. While the xenograft was clearly visualized in [^89^Zr]Mab#58 PET with apparently increasing radioactivity with time, the accumulation of [^89^Zr]IgG to the xenograft was low throughout the observed time period. Clear visualization of xenograft of other CTOSs derived from lung cancer specimen, in which HER3 was expressed, was also possible with [^89^Zr] Mab#58 (data not shown). Collectively, we demonstrated HER3 specific accumulation of [^89^Zr]Mab#58 in vivo and the potential of [^89^Zr]Mab#58 as a PET probe for non-invasive HER3 imaging not only in cancer cell line transplant model but also in more biologically and clinically relevant CTOS transplant models, in which the critical role of HER3 signaling was revealed.

When we compared PET images of [^89^Zr]Mab#58 to the control [^89^Zr]IgG of CTOS xenograft-bearing mice, focusing on the normal tissues, high kidney accumulation of [^89^Zr]IgG was notable. For ^89^Zr-labeled whole IgG, such a high accumulation in the kidney has rarely been reported [19, 29, 31, 35–37]. Because the control IgG and Mab#58 were of the same isotype and subclass, IgG_2a_, the difference would be mainly attributable to the characteristics of the individual antibody.

Among the HER family members, EGFR and HER2 have been very actively pursued as therapeutic targets in cancer. There have been numerous studies published on the development of EGFR and HER2 targeting molecules, such as antibodies and kinase inhibitors, for therapy as well as for imaging [38–40]. Compared to these, studies on HER3-targeting molecules have been few. As for PET or SPECT probes, only a few papers and abstracts have been published. These typically use antibodies and their fragments, owing mainly to the lack of kinase activity and, consequently, the lack of kinase inhibitors for HER3. These studies included ^99m^Tc-labeled affibody [18], ^89^Zr-labeled whole IgG [19], ^64^Cu-labeled F(ab′)2 [20] and ^64^Cu-labeled whole IgG [21]. They all reported the successful imaging of HER3 expressing tumors. The radioactivity found in the tumor varied, SUVs of 0.4–0.6 for F(ab)2, 2–4%ID/g for the affibody, and 10%–20% ID/g for whole IgG, and the non-specific accumulations and excretion routes were also varied, depending probably on the molecular size and the radioisotope and labeling method. The preferred combination of the molecule and the radioisotope would depend on the purpose, namely the assessment of HER3 expression or simulation and monitoring of in vivo behavior of the therapeutic antibodies, and also the properties of each antibody, especially their retention and clearance from the tissues. Further studies on non-invasive HER3 imaging, both in pre-clinical and clinical settings, would help to understand the in vivo properties of HER3, enabling a more efficient cancer treatment, not only those targeting HER family members but also chemotherapies through the formation of resistance which involves HER3.

CTOS xenografts have been reported to provide an in vivo model that maintains the properties of the original tumor of the patient from which the CTOS was prepared [22]. Furthermore, we previously demonstrated that the behavior of PET probes in CTOS xenograft model can reflect behavior observed in a clinical study [41]. The successful visualization of HER3 expression in CTOS xenografts by [^89^Zr]Mab#58 would support the applicability of antibody-based HER3-targeting to humans. Demonstration of the capability of the antibody to detect the levels of endogenous HER3 expression would further strengthen the notion, which would be in the scope of our future study. The present study, in which the target was found through the examination of CTOS, in addition to evaluation of the developed molecular probe using CTOS xenograft, could be perceived as an example of a new system. A system that could be used to develop pharmaceuticals with stronger relevance to the 3-dimensional nature of solid tumors and patient tumors than those commonly used with cell lines and cell line-based xenograft models.

## References

[1] Olayioye MA, Neve RM, Lane HA, Hynes NE. The ErbB signaling network: receptor heterodimerization in development and cancer. EMBO J. 2000 7 3; 19(13): 3159–3167. 10880430 10.1093/emboj/19.13.3159 PMC313958

[2] Hynes NE, Lane HA. ERBB receptors and cancer: the complexity of targeted inhibitors. Nat Rev Cancer 2005 5; 5(5): 341–354. 15864276 10.1038/nrc1609

[3] Normanno N, Bianco C, Strizzi L, Mancino M, Maiello MR, De Luca A, et al The ErbB receptors and their ligands in cancer: an overview. Curr Drug Targets. 2005 5; 6(3): 243–257. 15857286 10.2174/1389450053765879

[4] Gullick WJ. The c-erbB3/HER3 receptor in human cancer. Cancer Surv. 1996; 27: 339–349. 8909809

[5] Guy PM, Platko JV, Cantley LC, Cerione RA, Carraway KL 3rd. Insect cell-expressed p180erbB3 possesses an impaired tyrosine kinase activity. Proc Natl Acad Sci U S A. 1994 8 16; 91(17): 8132–8136. 8058768 10.1073/pnas.91.17.8132 PMC44559

[6] Sierke SL, Cheng K, Kim HH, Koland JG. Biochemical characterization of the protein tyrosine kinase homology domain of the ErbB3 (HER3) receptor protein. Biochem J. 1997 3 15; 322 (Pt 3): 757–763. 9148746 10.1042/bj3220757 PMC1218252

[7] Sliwkowski MX, Schaefer G, Akita RW, Lofgren JA, Fitzpatrick VD, Nuijens A, et al Coexpression of erbB2 and erbB3 proteins reconstitutes a high affinity receptor for heregulin. J Biol Chem. 1994 5 20; 269(20): 14661–14665. 7514177

[8] Hsieh AC, Moasser MM. Targeting HER proteins in cancer therapy and the role of the non-target HER3. Br J Cancer. 2007 8 20; 97(4): 453–457. 17667926 10.1038/sj.bjc.6603910 PMC2360352

[9] Tanner B, Hasenclever D, Stern K, Schormann W, Bezler M, Hermes M, et al ErbB-3 predicts survival in ovarian cancer. J Clin Oncol. 2006 9 10; 24(26): 4317–4323. 16896008 10.1200/JCO.2005.04.8397

[10] Witton CJ, Reeves JR, Going JJ, Cooke TG, Bartlett JM. Expression of the HER1-4 family of receptor tyrosine kinases in breast cancer. J Pathol. 2003 7; 200(3): 290–297. 12845624 10.1002/path.1370

[11] Engelman JA, Zejnullahu K, Mitsudomi T, Song Y, Hyland C, Park JO, et al MET amplification leads to gefitinib resistance in lung cancer by activating ERBB3 signaling. Science. 2007 5 18; 316(5827): 1039–1043. 17463250 10.1126/science.1141478

[12] Sergina NV, Rausch M, Wang D, Blair J, Hann B, Shokat KM, et al Escape from HER-family tyrosine kinase inhibitor therapy by the kinase-inactive HER3. Nature. 2007 1 25; 445(7126): 437–441. 17206155 10.1038/nature05474 PMC3025857

[13] Ma J, Lyu H, Huang J, Liu B. Targeting of erbB3 receptor to overcome resistance in cancer treatment. Mol Cancer. 2014 5 8; 13: 105 10.1186/1476-4598-13-105 24886126 PMC4022415

[14] Beji A, Horst D, Engel J, Kirchner T, Ullrich A. Toward the prognostic significance and therapeutic potential of HER3 receptor tyrosine kinase in human colon cancer. Clin Cancer Res. 2012 2 15; 18(4): 956–968. 10.1158/1078-0432.CCR-11-1186 22142822

[15] Lédel F, Hallström M, Ragnhammar P, Öhrling K, Edler D. HER3 expression in patients with primary colorectal cancer and corresponding lymph node metastases related to clinical outcome. Eur J Cancer. 2014 2; 50(3): 656–662. 10.1016/j.ejca.2013.11.008 24300455

[16] Kawakami H, Okamoto I, Yonesaka K, Okamoto K, Shibata K, Shinkai Y, et al The anti-HER3 antibody patritumab abrogates cetuximab resistance mediated by heregulin in colorectal cancer cells. Oncotarget. 2014 12 15; 5(23): 11847–11856. 25474137 10.18632/oncotarget.2663 PMC4323007

[17] van der Horst EH, Murgia M, Treder M, Ullrich A. Anti-HER-3 MAbs inhibit HER-3-mediated signaling in breast cancer cell lines resistant to anti-HER-2 antibodies. Int J Cancer. 2005 7 1; 115(4): 519–527. 15704104 10.1002/ijc.20867

[18] Orlova A, Malm M, Rosestedt M, Varasteh Z, Andersson K, Selvaraju RK, et al Imaging of HER3-expressing xenografts in mice using a (99m)Tc(CO) 3-HEHEHE-Z HER3:08699 affibody molecule. Eur J Nucl Med Mol Imaging. 2014 7; 41(7): 1450–1459. 10.1007/s00259-014-2733-7 24622956

[19] Terwisscha van Scheltinga AG, Lub-de Hooge MN, Abiraj K, Schröder CP, Pot L, Bossenmaier B, et al ImmunoPET and biodistribution with human epidermal growth factor receptor 3 targeting antibody ^89^Zr-RG7116. MAbs. 2014 Jul-Aug; 6(4): 1051–1058. 10.4161/mabs.29097 24870719 PMC4171008

[20] Wehrenberg-Klee E, Turker NS, Chang B, Heidari P, Mahmood U. Development of a HER3 PET probe for breast cancer imaging. J Nucl Med. 2014; 55 Suppl 1: 550.

[21] Sharp TL, Glaus C, Fettig N, Hewig A, Ogbagabriel S, Freeman D, et al Pharmacological evaluation of ^64^Cu-DOTA-AMG 888 (U3-1287) in control and tumor bearing mice using biodistribution and microPET imaging 2011 World Molecular Imaging Congress presentation number T206; 2011 9 7–11; San Diego, US.

[22] Kondo J, Endo H, Okuyama H, Ishikawa O, Iishi H, Tsujii M, et al Retaining cell-cell contact enables preparation and culture of spheroids composed of pure primary cancer cells from colorectal cancer. Proc Natl Acad Sci U S A. 2011 4 12; 108(15): 6235–6240. 10.1073/pnas.1015938108 21444794 PMC3076886

[23] Endo H, Okami J, Okuyama H, Kumagai T, Uchida J, Kondo J, et al Spheroid culture of primary lung cancer cells with neuregulin 1/HER3 pathway activation. J Thorac Oncol. 2013 2; 8(2): 131–139. 23328545 10.1097/JTO.0b013e3182779ccf

[24] Okuyama H, Yoshida T, Endo H, Nakayama M, Nonomura N, Nishimura K, et al Involvement of heregulin/HER3 in the primary culture of human urothelial cancer. J Urol. 2013 7; 190(1): 302–310. 10.1016/j.juro.2012.12.106 23313199

[25] Masuko T, Ohno Y, Masuko K, Yagi H, Uejima S, Takechi M, et al Towards therapeutic antibodies to membrane oncoproteins by a robust strategy using rats immunized with transfectants expressing target molecules fused to green fluorescent protein. Cancer Sci. 2011 1; 102(1): 25–35. 10.1111/j.1349-7006.2010.01741.x 21040216

[26] Ohno Y, Suda K, Masuko K, Yagi H, Hashimoto Y, Masuko T. Production and characterization of highly tumor-specific rat monoclonal antibodies recognizing the extracellular domain of human L-type amino-acid transporter 1. Cancer Sci. 2008 5; 99(5): 1000–1007. 10.1111/j.1349-7006.2008.00770.x 18294274 PMC11160021

[27] Ishimoto T, Oshima H, Oshima M, Kai K, Torii R, Masuko T, et al CD44+ slow-cycling tumor cell expansion is triggered by the cooperative actions of Wnt and prostaglandin E2 in gastric tumorigenesis. Cancer Sci. 2010 3; 101(3): 673–678. 10.1111/j.1349-7006.2009.01430.x 20028388 PMC11159848

[28] Yae T, Tsuchihashi K, Ishimoto T, Motohara T, Yoshikawa M, Yoshida GJ, et al Alternative splicing of CD44 mRNA by ESRP1 enhances lung colonization of metastatic cancer cell. Nat Commun. 2012 6 6; 3: 883 10.1038/ncomms1892 22673910

[29] Sugyo A, Tsuji AB, Sudo H, Nagatsu K, Koizumi M, Ukai Y, et al Evaluation of (89)Zr-labeled human anti-CD147 monoclonal antibody as a positron emission tomography probe in a mouse model of pancreatic cancer. PLoS One. 2013; 8(4): e61230 10.1371/journal.pone.0061230 23577210 PMC3618331

[30] Lindmo T, Boven E, Cuttitta F, Fedorko J, Bunn PA Jr. Determination of the immunoreactive fraction of radiolabeled monoclonal antibodies by linear extrapolation to binding at infinite antigen excess. J Immunol Methods. 1984 8 3;72(1):77–89. 6086763 10.1016/0022-1759(84)90435-6

[31] Perk LR, Vosjan MJ, Visser GW, Budde M, Jurek P, Kiefer GE, et al p-Isothiocyanatobenzyl-desferrioxamine: a new bifunctional chelate for facile radiolabeling of monoclonal antibodies with zirconium-89 for immuno-PET imaging. Eur J Nucl Med Mol Imaging. 2010 2; 37(2): 250–259. 10.1007/s00259-009-1263-1 19763566 PMC2816257

[32] Cushman SM, Jiang C, Hatch AJ, Shterev I, Sibley AB, Niedzwiecki D, et al Gene expression markers of efficacy and resistance to cetuximab treatment in metastatic colorectal cancer: results from CALGB 80203 (Alliance). Clin Cancer Res. 2015 3 1; 21(5): 1078–1086. 10.1158/1078-0432.CCR-14-2313 25520391 PMC4772749

[33] Wakui H, Yamamoto N, Nakamichi S, Tamura Y, Nokihara H, Yamada Y, et al Phase 1 and dose-finding study of patritumab (U3-1287), a human monoclonal antibody targeting HER3, in Japanese patients with advanced solid tumors. Cancer Chemother Pharmacol. 2014 3; 73(3): 511–516. 10.1007/s00280-014-2375-2 24442032 PMC3931937

[34] LoRusso P, Jänne PA, Oliveira M, Rizvi N, Malburg L, Keedy V, et al Phase I study of U3-1287, a fully human anti-HER3 monoclonal antibody, in patients with advanced solid tumors. Clin Cancer Res. 2013 6 1; 19(11): 3078–3087. 10.1158/1078-0432.CCR-12-3051 23591447

[35] Laverman P, van der Geest T, Terry SY, Gerrits D, Walgreen B, Helsen MM, et al Immuno-PET and immuno-SPECT of rheumatoid arthritis with radiolabeled anti-fibroblast activation protein antibody correlates with severity of arthritis. J Nucl Med. 2015 5; 56(5): 778–783. 10.2967/jnumed.114.152959 25858044

[36] Doran MG, Watson PA, Cheal SM, Spratt DE, Wongvipat J, Steckler JM, et al Annotating STEAP1 regulation in prostate cancer with 89Zr immuno-PET. J Nucl Med. 2014 12; 55(12): 2045–2049. 10.2967/jnumed.114.145185 25453051 PMC4410715

[37] Kuo F, Histed S, Xu B, Bhadrasetty V, Szajek LP, Williams MR, et al Immuno-PET imaging of tumor endothelial marker 8 (TEM8). Mol Pharm. 2014 11 3; 11(11): 3996–4006. 10.1021/mp500056d 24984190 PMC4224515

[38] Iqbal N, Iqbal N. Human epidermal growth factor receptor 2 (HER2) in cancers: overexpression and therapeutic implications. Mol Biol Int. 2014; 2014: 852748 10.1155/2014/852748 25276427 PMC4170925

[39] Cai W, Niu G, Chen X. Multimodality imaging of the HER-kinase axis in cancer. Eur J Nucl Med Mol Imaging. 2008 1; 35(1): 186–208. 17846765 10.1007/s00259-007-0560-9

[40] Corcoran EB, Hanson RN. Imaging EGFR and HER2 by PET and SPECT: a review. Med Res Rev. 2014 5; 34(3): 596–643. 10.1002/med.21299 24037872

[41] Furukawa T, Yuan Q, Jin ZH, Aung W, Yoshii Y, Hasegawa S, et al Comparison of intratumoral FDG and Cu-ATSM distributions in cancer tissue originated spheroid (CTOS) xenografts, a tumor model retaining the original tumor properties. Nucl Med Biol. 2014 9; 41(8): 653–659. 10.1016/j.nucmedbio.2014.05.139 24997088

